# The grit effect: predicting retention in the military, the workplace, school and marriage

**DOI:** 10.3389/fpsyg.2014.00036

**Published:** 2014-02-03

**Authors:** Lauren Eskreis-Winkler, Elizabeth P. Shulman, Scott A. Beal, Angela L. Duckworth

**Affiliations:** ^1^Department of Psychology, University of PennsylvaniaPhiladelphia, PA, USA; ^2^Fort Bragg Research Element, U.S. Army Research InstituteFort Belvoir, VA, USA

**Keywords:** grit, conscientiousness, personality, retention, dropout

## Abstract

Remaining committed to goals is necessary (albeit not sufficient) to attaining them, but very little is known about domain-general individual differences that contribute to sustained goal commitment. The current investigation examines the association between grit, defined as passion and perseverance for long-term goals, other individual difference variables, and retention in four different contexts: the military, workplace sales, high school, and marriage. Grit predicted retention over and beyond established context-specific predictors of retention (e.g., intelligence, physical aptitude, Big Five personality traits, job tenure) and demographic variables in each setting. Grittier soldiers were more likely to complete an Army Special Operations Forces (ARSOF) selection course, grittier sales employees were more likely to keep their jobs, grittier students were more likely to graduate from high school, and grittier men were more likely to stay married. The relative predictive validity of grit compared to other traditional predictors of retention is examined in each of the four studies. These findings suggest that in addition to domain-specific influences, there may be domain-general individual differences which influence commitment to diverse life goals over time.

## Introduction

In his advice to aspiring writers, Woody Allen famously quipped “80% of success is showing up” (Safire, 1989, p. A10). As Allen elaborated, “My observation was that once a person actually completed a play or a novel, he was well on his way to getting it produced or published, as opposed to a vast majority of people who tell me their ambition is to write, but who strike out on the very first level and indeed never write the play or book” (p. A10). The importance of goal commitment to performance seems obvious on logical grounds. Giving up on a goal is, as Allen put it, striking out on the very first level. It guarantees failure. The current investigation asks whether there are individual differences that predict showing up across diverse life contexts.

Across work, school, and marital contexts there is variation in the degree to which people “show up” and keep showing up. Approximately 50% of soldiers enrolled in the U.S. Army's Special Operations Forces selection course drop out during the selection process (Bartone et al., 2008). Even in less physically demanding work settings, such as workplace sales, turnover hovers around 50% (Landau and Werbel, 1995; Richardson, 1999). Nationally, 25% of students drop out of school before earning their high school diplomas, and dropout among students from disadvantaged minority backgrounds is twice that figure (Swanson, 2004). Likewise, despite the hopeful newlywed promise “til death do us part,” approximately half of American marriages end in divorce (Schoen and Standish, 2001; Raley and Bumpass, 2003; Stevenson and Wolfers, 2007). Military dropout, workplace turnover, high school dropout and divorce are typically studied in isolation of one another, an uncoordinated approach that would seem profitable insofar as the determinants of dropout differ by context. However in addition to domain-specific factors, personality traits that capture general dispositions may also be relevant. The hypothesis of the current investigation is that grit, the tendency to sustain passion and perseverance for long-term goals, is a domain-general trait that promotes “showing up” across diverse life contexts. Across four different life contexts, we examine the relative predictive validity of grit to determine whether it explains retention over and beyond established domain-specific predictors of retention and other individual differences.

## Past research

In examining census data from the 1950's, the demographer Paul Glick noted that high school and college dropouts had significantly higher divorce rates than the general population (Glick and Carter, 1958)—a phenomenon later dubbed “The Glick Effect” (Bauman, 1967). Glick hypothesized that “certain predisposing factors in the … psychological orientation of these persons [affect] persistence in education [and] persistence in marriage” (Glick and Carter, 1958, p. 154). However in the years after Glick's speculation, the tide of psychological research shifted toward the study of situational as opposed to domain-general determinants of human behavior (Mischel, 1968). The importance of specific situational factors does not rule out the possibility that traits too can influence behavior (Roberts, 2009). The current investigation revisits Glick's hypothesis, examining the association between grit, a psychological trait, and dropout across work, school, and marital contexts.

Grit is the disposition to pursue long-term goals with sustained interest and effort over time (Duckworth et al., 2007). The notion that sustained effort and focused interests are distinct from talent but equally vital to success has been discussed in the psychological literature for well over a century. In perhaps the earliest systematic inquiry into the psychological determinants of high achievement, Galton (1892) reviewed biographical information on prominent individuals in an array of disciplines including science, poetry, music, art, and the law. Galton proposed that talent was insufficient for eminent achievement. Rather, the most eminent individuals displayed ability combined with “zeal,” and the “capacity for hard labour” (p. 38).

Recent research supports Galton's intuition. Grit is associated with lifetime educational attainment (Duckworth et al., 2007). Grit predicts teacher effectiveness (Duckworth et al., 2009; Robertson-Kraft and Duckworth, in press), academic performance at elite universities (Duckworth et al., 2007), and final rank in the National Spelling Bee (Duckworth et al., 2007, 2011). Of particular relevance to the current investigation, West Point cadets one standard deviation higher in grit have 62% higher odds of remaining at West Point long-term. Notably, grit more strongly predicts cadet retention than does SAT score, high school rank, or self-control (Duckworth et al., 2007).

The psychological construct of grit is one facet of Big Five conscientiousness (Duckworth et al., 2007), a broad family of personality traits that includes many other facets (e.g., self-discipline, dutifulness, achievement striving). Both conscientiousness, the facets of conscientiousness, and other related constructs (e.g., self-control, low impulsivity, discipline) demonstrate positive associations with achievement (Valiente et al., 2008, 2010; Poropat, 2009) and negative associations with high school and college dropout (Kelly and Veldman, 1964; Robbins et al., 2004). Unlike other facets of conscientiousness, grit denotes extreme stamina in terms of particular interests and applied effort toward these interests. Grit is not just about working hard on tasks at hand but, rather, working diligently toward the same higher-order goals over extremely long stretches of time. In line with the hypotheses of Paunonen and Ashton (2001), grit, a narrow facet of conscientiousness, has demonstrated incremental predictive validity over and above Big Five conscientiousness for achievement outcomes (Duckworth et al., 2007).

## Current investigation

With the exception of the West Point study mentioned above, the association between grit and retention has not been examined. In the current investigation, we assess the predictive validity of grit for retention, alongside traditionally established predictors of retention, in four life domains. Studies 1, 2 and 3, using longitudinal designs, assess the extent to which grit and other variables prospectively predict program completion among 677 soldiers in an Army Special Operations Forces (ARSOF) selection course, job retention among 442 sales representatives at a vacation ownership corporation, and on-time graduation among 4813 juniors in the Chicago public high schools. Study 4, a cross-sectional study, assesses the association between grit, Big Five personality traits, and marital status in an Internet sample of over 6000 adults. In all four studies, we estimate the unique variance in retention explained by grit alongside other individual difference variables and demographic variables. The Institutional Review Board at the University of Pennsylvania approved all studies in this paper. All participants were consented prior to their participation in these studies.

## Study 1

In Study 1, we examine the extent to which grit predicts completion of a grueling, 24-day Army Special Operations Forces (ARSOF) selection course. Among other challenges, ARSOF candidates complete time-limited land navigation courses carrying heavy loads of equipment, with no assistance from instructors or fellow students. Only graduates of a 30-day Special Operations Preparation Course are qualified to begin this ARSOF selection course. Even with this stringent entrance requirement, approximately half of ARSOF candidates voluntarily withdraw before the end of the 24-day selection course (Bartone et al., 2008). Our aim in Study 1 was to examine the predictive validity of grit, measured at the start of ARSOF selection, for successful course completion. Analyses in Study 1 controlled for demographic characteristics as well as general intelligence and physical fitness, two established predictors of attrition in military settings (Burke et al., 1989; Pope et al., 1999; National Research Council, 2006; Niebuhr et al., 2008).

### Materials and methods

#### Participants

Participants were members of four consecutive cohorts admitted to ARSOF selection courses between November 2008 and February 2009. We excluded from our final sample 12% of the original 824 candidates who withdrew for medical reasons (e.g., injury or pre-existing medical conditions) as well as the 1% of candidates who had incomplete data^1^. The final sample, *N* = 677 (82%), was typical of recent selection classes in terms of gender (100% male) and age (*M* = 25.61 years, *SD* = 4.39). Years of schooling was the only variable on which included participants differed from excluded participants [*t*_(193)_ = 3.52, *p* < 0.01, *d* = 0.34], with included participants reporting slightly less formal schooling (*M* = 12.98, *SD* = 1.88) than excluded participants (*M* = 13.65, *SD* = 2.11).

#### Procedure and measures

Candidates were evaluated on their general intelligence, physical fitness, and grit prior to the start of the 24-day training regimen.

***Retention***. Candidates who completed ARSOF were coded as 1 = *retained*; candidates who voluntarily withdrew were coded as 0 = *dropped out*.

***Grit***. Grit was assessed with the eight-item Short Grit Scale (Duckworth and Quinn, 2009). Participants endorsed items describing their tendency to maintain effort (e.g., “Setbacks don't discourage me”) and focused interest (e.g., “I have been obsessed with a certain idea or project for a short time but later lost interest,” reverse scored) using a 5-point Likert-like scale from 1 = *not at all like me* to 5 = *very much like me*. The observed alpha was 0.77.

***General intelligence***. Participants completed the Armed Services Vocational Aptitude Battery—General Technical (ASVAB-GT; McLaughlin et al., 1984), which evaluates cognitive ability and includes tests of arithmetic reasoning, word knowledge, and paragraph comprehension.

***Physical fitness***. The Army Physical Fitness Test assesses candidates on push-ups, sit-ups and a timed two-mile run, each of which is scored on a 0–100 scale for a maximum attainable total score is 300.

***Years of schooling***. Candidates self-reported how many years of schooling they completed prior to the start of training.

### Statistical analyses

In the current study and the studies that follow, we first examined bivariate associations between predictor variables and retention in separate binary logistic regression models. Next, to assess the incremental predictive validity of grit, we fit a full logistic regression model predicting retention from grit, any individual difference variables that demonstrated significant bivariate associations with retention, and all available demographic variables. Finally, we conducted a hierarchical logistic regression to determine whether grit predicts unique variance in retention over and beyond all other individual difference variables and demographic variables in the full model.

Continuous predictors were standardized to yield a more intuitive interpretation of odds ratios (*OR*). Reported *OR*s represent a change in the odds of retention for a one standard deviation change in the predictor variable. Variance inflation factor (VIF) scores were below 2.0 for all independent variables across studies, suggesting that multicollinearity was not a problem (Ryan, 1997).

### Results and discussion

Fifty-eight percent of candidates successfully completed ARSOF selection. As shown in Table 1A, grit was not correlated with either general intelligence or physical fitness.

**Table 1A:** **Summary statistics and intercorrelations among ARSOF candidates (Study 1)**.

**Variables**	**Correlations^a^**		
	**1**	**2**	**3**
1. Grit			
2. General intelligence	−0.07		
3. Physical fitness	0.06	0.09^*^	
4. Age	0.12^**^	−0.05	−0.13^**^
5. Years of schooling	−0.00	0.42^***^	0.11^**^
Observed range	2.00–5.00	100–149	166–300
*M*	3.97	115.75	257.78
*SD*	0.51	9.27	26.26

N = 677.

* p < 0.05;

** p < 0.01;

*** p < 0.001.

a Full correlations among demographics are truncated to conserve space.

As shown in Table 1B, in separate binomial logistic regression models, grit (*OR* = 1.28), general intelligence (*OR* = 1.60) and physical fitness (*OR* = 1.79) predicted retention. Years of schooling (*OR* = 1.53) had a significant bivariate association with retention whereas age did not. Because grit was independent of both general intelligence and physical fitness, it was not surprising that in a full model controlling for general intelligence, physical fitness, age and years of schooling, the effect of grit (*OR* = 1.32) remained significant: Candidates one standard deviation higher in grit had 32% higher odds of completing ARSOF selection.

**Table 1B:** **Bivariate and full logistic regressions predicting ARSOF retention (Study 1)**.

**Measures**	**Bivariate model**			**Full model**		
	***OR***	**95% CI**	**% *R*^2^^a^**	***OR***	**95% CI**	**% *R*^2^^a^**
Grit	1.28^**^	[1.09, 1.49]	1.92	1.32^**^	[1.12, 1.56]	1.84
General intelligence	1.60^***^	[1.35, 1.89]	6.41	1.46^***^	[1.20, 1.76]	2.67
Physical fitness	1.79^***^	[1.52, 2.11]	10.14	1.72^***^	[1.45, 2.04]	7.28
Age	0.94	[0.80, 1.10]	0.12	0.93	[0.77, 1.12]	0.09
Years of schooling	1.53^***^	[1.28, 1.82]	4.86	1.31^*^	[1.07, 1.60]	1.19

N = 677.

* p < 0.05;

** p < 0.01;

*** p < 0.001.

a The Nagelkerke index was used to compute Pseudo R^2^.

Grit (Nagelkerke Δ*R*^2^ = 1.84%), general intelligence (Nagelkerke Δ*R*^2^ = 2.67%), and physical fitness (Nagelkerke Δ*R*^2^ = 7.28%) explained unique variance in the retention outcome (see Table 1B). We next ran a hierarchical logistic regression to confirm the unique predictive validity of grit over and above all other predictors in the model. All predictor variables except grit were entered in Step 1 of this model. Grit was entered in Step 2, as shown in Table 1B. The results of this hierarchical logistic regression revealed that Step 2 contributed significantly to the model, χ^2^_(1)_ = 10.65, *p* < 0.001.

Overall, the results indicated that gritty individuals were less likely to voluntarily drop out of an arduous 24-day ARSOF selection course. Notably, the effect of grit on retention held when controlling for general intelligence and physical fitness, the army's traditional predictors of retention.

## Study 2

In Study 1, grit predicted successful completion of an elite military selection program over and above general intelligence and physical fitness. In Study 2, we examined whether grit also predicts retention among sales representatives at a vacation ownership corporation.

Prior studies have found that retention in sales jobs is predicted by Big Five emotional stability, conscientiousness, agreeableness and, to a lesser degree, by extraversion and (inversely) openness to experience (Mobley et al., 1979; Steers and Mowday, 1981; Costa and McCrae, 1985; Salgado, 2002; Zimmerman, 2008). Demographic variables (e.g., age) are also strongly associated with retention (Porter and Steers, 1973; Price, 1977; Muchinsky and Tuttle, 1979; Muchinsky and Morrow, 1980; Ladik et al., 2002). Thus, in this prospective, longitudinal study we measured the predictive validity of grit for retention in sales, as well as the predictive validity of Big Five personality traits, while controlling for relevant demographic variables.

### Materials and methods

#### Participants

We distributed consent forms and questionnaires to 1104 sales representatives at six different sites of a vacation ownership corporation. Of 714 sales representatives who returned surveys, 62% (*N* = 442) were complete. Seventy-seven percent of participants were White, 9% were Black, 7% were Hispanic, 2% were Asian, and 5% were of other ethnic backgrounds; 61% of were male (*M* = 43.97 years, *SD* = 11.86). On average, employees had over a decade of experience in sales (*M* = 12.34 years; *SD* = 10.73). No significant differences between included vs. excluded participants emerged on any measured variables (*p*s > 0.05).

#### Procedure and measures

Consent forms and questionnaires were distributed in July 2007 and retention was assessed in January 2008, 6 months later.

***Retention***. We coded 1 = *retained* for participants who had a fall sales record (August through January), and 0 = *dropped out* for participants with no sales on record for the fall period (August through January). Although coding retention in this way was only a rough approximation of true retention since it may have erroneously classed individuals on maternity leave, sick leave, or other temporary leave as dropouts, it was recommended by the vacation ownership corporation as the most accurate way to capture retention based on company records.

***Grit***. As in Study 1, participants completed the Short Grit Scale (Duckworth and Quinn, 2009). The observed alpha was 0.79.

***Big Five personality traits***. Participants endorsed the 44-item Big Five Inventory (BFI; see Benet-Martínez and John, 1998). Participants endorsed each item (e.g., “I am very motivated by making money,” and, “I see myself as someone who is talkative.”) on a 5-point scale ranging from 1 = *disagree strongly* to 5 = *agree strongly.* Observed alphas ranged from 0.75 to 0.82.

***Weeks employed and years of sales experience***. The number of weeks each participant had been employed by the corporation was obtained from human resource records. Participants self-reported the total number of years they had worked in sales. Both variables were right-skewed, so we employed natural log transformations to normalize these distributions.

***Site***. Participants self-reported site placement (at one of six office sites).

### Results and discussion

Forty-five percent of sales employees were retained at follow-up in January 2008. Consistent with prior studies (e.g., Duckworth et al., 2007), grit was strongly associated with Big Five conscientiousness (*r* = 0.64) and more weakly associated with other Big Five traits (*r*s from 0.19 to 0.48) (see Table 2A).

**Table 2A:** **Summary statistics and intercorrelations for sales employees (Study 2)**.

**Variables**	**Correlations^a^**					
	**1**	**2**	**3**	**4**	**5**	**6**
1. Grit	–					
2. Big Five extraversion	0.25^***^	–				
3. Big Five agreeableness	0.39^***^	0.26^***^	–			
4. Big Five conscientiousness	0.64^***^	0.37^***^	0.53^***^	–		
5. Big Five emotional stability	0.48^***^	0.30^***^	0.44^***^	0.53^***^	**–**	
6. Big Five openness to experience	0.19^***^	0.34^***^	0.30^***^	0.33^***^	0.31^***^	–
7. Female	−0.02	0.09	0.11^*^	0.05	−0.08	0.05
8. Age	0.20^***^	0.00	0.15^**^	0.17^***^	0.21^***^	0.02
9. White	−0.10^*^	0.07	−0.01	−0.02	−0.15^**^	−0.09
10. Black	0.10^*^	−0.06	0.01	0.01	0.12^*^	0.04
11. Hispanic	−0.01	−0.03	−0.01	−0.02	0.08	0.07
12. Asian	0.04	−0.03	0.02	0.04	0.02	0.04
13. Other	0.03	−0.01	0.02	0.03	−0.01	0.04
14. Weeks employed	0.08	0.03	−0.01	0.06	−0.05	−0.02
15. Years in sales	0.15^**^	0.08	0.08	0.12^*^	0.09	0.06
Observed range	1.50–5.00	2.25–5.00	2.44–5.00	2.38–5.00	1.88–5.00	2.20–5.00
*M*	4.20	3.98	4.13	4.15	3.82	3.93
*SD*	0.63	0.58	0.47	0.52	0.62	0.51

N = 442.

* p < 0.05;

** p < 0.01;

*** p < 0.001.

a Full correlations among demographics are truncated to conserve space.

As shown in Table 2B, in separate binomial logistic regression models, among personality variables only grit (*OR* = 1.38) predicted retention. Demographic and background variables such as age, gender, race, and site were not associated with retention, whereas weeks employed (*OR* = 2.78) and years in sales (*OR* = 1.21) were positively associated with retention. Because there was a trend toward conscientiousness (*OR* = 1.20, *p* = 0.06) predicting retention, we fit a full model to test the incremental predictive validity of grit, controlling for conscientiousness and all demographic variables. The effect of grit, in this final model, remained significant (*OR* = 1.40): candidates one standard deviation higher in grit had 40% higher odds of workplace retention.

**Table 2B:** **Bivariate and full logistic regressions predicting sales employee retention (Study 2)**.

**Variables**	**Bivariate model**			**Full model**		
	***OR***	**95% CI**	***% R*^2^^a^**	***OR***	**95% CI**	***% R*^2^^a^**
Grit	1.38^**^	[1.14, 1.67]	3.30	1.40^*^	[1.05, 1.87]	1.27
Big Five extraversion	1.14	[0.94, 1.37]	0.54	–	–	–
Big Five agreeableness	1.10	[0.90, 1.33]	0.26	–	–	–
Big Five conscientiousness	1.20^†^	[0.99, 1.45]	1.09	1.03	[0.77, 1.37]	0.00
Big Five emotional stability	1.03	[0.86, 1.24]	0.03	–	–	–
Big Five openness to experience	0.95	[0.79, 1.15]	0.07	–	–	–
Female	0.74	[0.50, 1.09]	0.72	0.75	[0.47, 1.17]	0.37
Age	0.89	[0.73, 1.07]	0.49	0.65^**^	[0.50, 0.84]	2.58
Black	1.05	[0.54, 2.03]	0.00	1.04	[0.45, 2.38]	0.00
Hispanic	1.06	[0.50, 2.23]	0.00	0.79	[0.34, 1.83]	0.07
Asian	4.92	[0.59, 41.22]	0.90	2.70	[0.29, 24.94]	0.21
Other	2.02	[0.82, 4.98]	0.76	2.28	[0.84, 6.15]	0.64
Weeks employed	2.78^***^	[2.18, 3.55]	23.47	2.94^***^	[2.26, 3.83]	20.17
Years in sales	1.21^*^	[1.01, 1.46]	1.27	1.32^*^	[1.02, 1.70]	1.06

N = 442.

* p < 0.05;

** p < 0.01;

*** p < 0.001;

† p = 0.06.

a The Nagelkerke index was used to compute Pseudo R^2^.

Grit explained unique variance in the retention outcome (Nagelkerke Δ*R*^2^ = 1.27%) whereas conscientiousness did not (Nagelkerke Δ*R*^2^ = 0.00%) (see Table 2B). Weeks employed (Nagelkerke Δ*R*^2^ = 20.17%) and years in sales (Nagelkerke Δ*R*^2^ = 1.06%) were the only background variables to reach significance in the final model. We next ran a hierarchical logistic regression to confirm the unique predictive validity of grit over and above all other predictors. All predictor variables except grit were entered in Step 1 of this model. Grit was entered in Step 2, as shown in Table 2B. Results of the hierarchical logistic regression revealed that Step 2 (grit) contributed significantly to the model, χ^2^_(1)_ = 6.38, *p* < 0.012.

Overall, the results indicated that gritty sales representatives were more likely to remain at their jobs long-term. Notably, the effect of grit on retention held when controlling for Big Five conscientiousness and demographic variables.

## Study 3

In Study 3, we examined the predictive validity of grit for graduation from the Chicago Public Schools. Established demographic predictors of high school graduation include race (Jordan et al., 1996), gender (Jordan et al., 1996; Swanson, 2004), and socioeconomic status (Rosenthal, 1998). Graduation is also strongly predicted by situational factors, including school safety (DeLuca and Rosenbaum, 2000), teacher support (Catterall, 1998; Croninger and Lee, 2001; Lee and Burkam, 2003; Brewster and Bowen, 2004), parental support (McNeal, 1999), and peer support (Kasen et al., 1998). Individual differences, including conscientiousness (Okun and Finch, 1998; Tross et al., 2000) and intelligence (Jimerson et al., 2000), have also been shown to predict high school graduation. In Study 3, we examined whether students' grit, measured junior year, predicted graduation senior year from the Chicago Public Schools. In Study 3, as in prior studies, our analyses controlled for established demographic, situational, and individual difference variables.

### Materials and methods

#### Participants

Participants were high school juniors in 98 Chicago Public Schools who completed a survey administered by the Chicago Consortium on School Research at the close of the 2008–2009 academic year. Although 12,198 juniors took the administered survey, the final sample had *N* = 4813 students (39%) after excluding students who were either missing survey responses or basic demographic data. Approximately 45% of participants were Hispanic, 43% were Black, 6% were White, 5% were Asian, and 1% were of other ethnic backgrounds; 58% were male. Differences between included and excluded participants were statistically significant (due to the large sample) but small in magnitude for all variables (*d*s < 0.2 for continuous variables and ϕs < 0.14 for categorical variables).

#### Procedure and measures

Participants completed self-report questionnaires during the regular school day in the spring of their junior year (see Consortium on Chicago School Research, 2009 for details regarding survey administration). The survey included 47 scales (193 items total) developed by teachers and principals through an “extensive stakeholder consultation and review process” (Consortium on Chicago School Research, 2009). Seven of the 47 scales with theoretical relevance to high school retention were considered as covariates. Internal reliabilities of included scales ranged from 0.83 to 0.91. Excluded scales asked students to rate the quality of their classes (e.g., “This class really makes me think,” “In class we build off each other's ideas,” “In science class this year, we often wrote lab reports”) as well as the content of specific classes, such as how often in science class they were asked to write lab reports.

***Retention: high school graduation***. Available school records noted a positive graduation status for students who graduated in spring 2010, but did not have any detailed information about students without confirmed graduation status. (It was unknown whether these students dropped out, transferred to another school district, etc.) We coded students with confirmed graduation status as 1 = *retained* and coded students without confirmed graduation status as 0 = *dropped out.*

***Grit***. Participants endorsed four items from the Short Grit Scale (Duckworth and Quinn, 2009) using a 5-point Likert-type scale from 1 = *not at all like me* to 5 = *very much like me*. The phrasing of two items was simplified in anticipation of the reading level of younger participants: “I don't give up easily” was substituted for the original scale's “Setbacks don't discourage me,” and “I continue steadily towards my goals” was substituted for “I often set a goal but later choose to pursue a different one.” The observed alpha of the four-item measure was 0.90.

***Academic conscientiousness and school motivation***. Participants endorsed nine items (e.g., “I set aside time to do my homework and study.”) describing conscientious academic behaviors on a 4-point scale ranging from 1 = *strongly disagree* to 4 = *strongly agree*. The observed alpha was.91. In addition, participants endorsed five school motivation items (e.g., “My classes give me useful preparation for what I plan to do in life.”) on a 4-point scale ranging from 1 = *strongly disagree* to 4 = *strongly agree*. The observed alpha was 0.90.

***Situational factors***. Participants endorsed eight items about the safety of their particular school (e.g., “I worry about crime and violence in school.”) on a 4-point scale ranging from 1 = *not safe* to 4 = *very safe*. Participants endorsed twelve items about how supportive they perceived their teachers to be (e.g., “Teachers work hard to make sure that students stay in school.”) on a 4-point scale ranging from 1 = *strongly disagree* to 4 = *strongly agree*. Participants endorsed three items about how supportive they perceived their parents to be (e.g., “This year my parents/guardians have talked to me about how I am doing in my classes”) on a 4-point scale ranging from 1 = *never* to 4 = *frequently*. Finally, participants endorsed six items about peer support (e.g., “My friends and I help each other prepare for tests”) on a 4-point scale ranging from 1 = *strongly disagree* to 4 = *strongly agree.* The observed alphas of the *school safety*, *teacher support*, *parent support*, and *peer support* scales were 0.84, 0.93, 0.91, and 0.91, respectively.

***Standardized achievement test scores***. The Prairie State Achievement Examination, taken by all high school juniors, evaluates reading and math skills. Math and reading scores were highly correlated (*r* = 0.75), so we averaged them to create a composite standardized achievement test score for each student.

***Socioeconomic status***. Free lunch status was used as a proxy for socioeconomic status. All students had one of three possible lunch statuses: full price lunch, reduced-price lunch, or free lunch. Students with free lunch eligible status come from families with incomes at or below 130% of the federal poverty level (for the period from July 1, 2008 through June 30, 2009, 130% of the poverty level was $27,560 for a family of four). Students eligible for reduced lunch come from families with incomes between 130 and 185% of the poverty level (for the period from July 1, 2008 through June 30, 2009, 185% of the poverty level was $39,220 for a family of four; United States Department of Agriculture, Food and Nutrition Services, 2013).

### Results and discussion

Eighty-eight percent of Chicago Public School students surveyed in the spring of their junior year graduated 1 year later. As reported in Table 3A, grit was strongly correlated with both academic conscientiousness (*r* = 0.49) and school motivation (*r* = 0.49).

**Table 3A:** **Summary statistics and intercorrelations for Chicago public school students (Study 3)**.

**Measures**	**Correlations^a^**							
	**1**	**2**	**3**	**4**	**5**	**6**	**7**	**8**
1. Grit	–							
2. Standardized achievement tests	0.15^***^	–						
3. Academic conscientiousness	0.49^***^	0.06^***^	–					
4. School motivation	0.49^***^	0.12^***^	0.50^***^	–				
5. Perceived school safety	0.15^***^	0.28^***^	0.13^***^	0.16^***^	–			
6. Perceived teacher support	0.38^***^	0.12^***^	0.51^***^	0.55^***^	0.26^***^	–		
7. Perceived parental support	0.34^***^	0.07^***^	0.29^***^	0.30^***^	0.10^***^	0.27^***^	–	
8. Perceived peer support	0.42^***^	0.13^***^	0.48^***^	0.53^***^	0.19^***^	0.53^***^	0.27^***^	–
9. Female	0.14^***^	−0.01	0.13^***^	0.14^***^	−0.08^***^	0.09^***^	0.06^***^	0.15^***^
10. Hispanic	−0.08^***^	0.04^*^	−0.09^***^	−0.03^*^	0.02	0.00	−0.05^**^	−0.07^***^
11. Black	0.07^***^	−0.20^***^	0.06^***^	0.03	−0.11^***^	−0.03	0.06^***^	0.06^***^
12. White	0.00	0.15^***^	0.02	−0.01	0.10^***^	0.00	0.02	−0.03
13. Asian	0.02	0.20^***^	0.05^**^	0.03^*^	0.09^***^	0.06^***^	−0.05^**^	0.04^**^
14. Other	0.03^*^	0.01	0.01	0.01	0.02	0.00	−0.00	0.01
15. Free lunch	−0.01	−0.18^***^	−0.02	−0.01	−0.07^***^	−0.03^*^	−0.03	−0.03
16. Reduced price lunch	0.00	0.09^***^	0.02	0.01	0.03^*^	0.01	0.01	0.01
17. Full price lunch	−0.00	0.15^***^	0.01	−0.01	0.07^***^	0.03^*^	0.02	0.02
Observed range	1.00–5.00	120.50–198.50	1.00–4.00	1.00–4.00	1.00–4.00	1.00–4.00	1.00–4.00	1.00–4.00
*M*	3.89	149.46	2.80	3.11	2.77	2.81	2.99	2.83
*SD*	0.89	12.45	0.56	0.64	0.58	0.55	0.89	0.61

N = 4813.

* p < 0.05;

** p < 0.01;

*** p < 0.001.

a Full correlations among demographics are truncated to conserve space.

As shown in Table 3B, in separate binomial logistic regression models, high school graduation was predicted by grit (*OR* = 1.48), as well as academic conscientiousness (*OR* = 1.31), school motivation (*OR* = 1.40), and standardized achievement test scores (*OR* = 1.95), all measured during junior year. Situational factors also predicted high school graduation (effect sizes ranged from *OR* = 1.20 for perceived school safety to *OR* = 1.38 for perceived teacher support). Demographic characteristics associated with retention in bivariate analyses were gender, race and SES: Females (*OR* = 2.05), Asians (*OR* = 5.30), and students who received reduced price lunch (*OR* = 1.47) were more likely to graduate, whereas students who received free lunch (*OR* = 0.68) were less likely to graduate.

**Table 3B:** **Bivariate and full logistic regressions predicting high school retention (Study 3)**.

**Measures^a^**	**Bivariate model**			**Full model**		
	***OR***	**95% CI**	**% *R*^2^^a^**	***OR***	**95% CI**	**% *R*^2^^a^**
Grit	1.48^***^	[1.37, 1.61]	3.40	1.21^***^	[1.09, 1.34]	0.50
Academic conscientiousness	1.31^***^	[1.20, 1.42]	1.50	1.00	[0.89, 1.13]	0.00
School motivation	1.40^***^	[1.29, 1.52]	2.42	1.07	[0.95, 1.21]	0.05
Perceived school safety	1.20^***^	[1.10, 1.31]	0.74	1.04	[0.94, 1.14]	0.02
Perceived teacher support	1.38^***^	[1.27, 1.50]	2.17	1.14^*^	[1.01, 1.29]	0.17
Perceived parental support	1.21^***^	[1.11, 1.31]	0.77	1.00	[0.91, 1.11]	0.00
Perceived peer support	1.32^***^	[1.22, 1.43]	1.66	0.97	[0.86, 1.09]	0.01
Standardized achievement tests	1.95^***^	[1.75, 2.17]	6.46	1.78^***^	[1.60, 2.00]	4.03
Female	2.05^***^	[1.73, 2.44]	2.62	1.92^***^	[1.60, 2.30]	1.83
Black	0.96	[0.81, 1.14]	0.00	1.16	[0.96, 1.40]	0.08
White	1.03	[0.73, 1.46]	0.00	0.85	[0.58, 1.24]	0.03
Asian	5.30^***^	[2.34, 11.98]	1.09	3.34^**^	[1.45, 7.69]	0.42
Other	0.58	[0.06, 5.15]	0.00	0.30	[0.03, 2.71]	0.03
Free lunch	0.68^**^	[1.11, 1.31]	0.48	0.92	[0.64, 1.33]	0.01
Reduced price lunch	1.47^**^	[1.12, 1.95]	0.32	1.25	[0.81, 1.95]	0.04

N = 4813.

* p < 0.05;

** p < 0.01;

*** p < 0.001.

a The Nagelkerke index was used to compute Pseudo R^2^.

In a binary logistic regression model controlling for all measured individual difference variables and situational variables, as well as standardized achievement test scores and demographic covariates, grit remained a significant predictor of graduation (*OR* = 1.21). Students one standard deviation higher in grit their junior year had 21% higher odds of graduating from high school on time.

Examining the relative predictive validity of grit compared to other individual difference variables in this final model revealed that, among non-demographic variables, only grit (Nagelkerke Δ*R*^2^ = 0.50%), perceived teacher support (Nagelkerke Δ*R*^2^ = 0.17%), standardized achievement test scores (Nagelkerke Δ*R*^2^ = 4.03%), being female (Nagelkerke Δ*R*^2^ = 1.83%), and being Asian (Nagelkerke Δ*R*^2^ = 0.42%) explained unique variance in retention. Subsequent to this full model, we ran a hierarchical logistic regression to confirm the unique predictive validity of grit over and above all other predictors. All predictor variables except grit were entered in Step 1 of this model. Grit was entered in Step 2, as shown in Table 3B. Results of this hierarchical logistic regression revealed that Step 2 contributed significantly to the model, χ^2^_(1)_ = 16.17, *p* < 0.001.

Overall, the results indicated that gritty juniors were more likely to graduate from high school their senior year. Notably, the effect of grit on retention held when controlling for academic conscientiousness, school motivation, situational factors, standardized achievement test scores, and demographic variables.

## Study 4

In contrast to the three preceding studies which examined retention in traditional achievement domains, in Study 4 we examined the association between grit and the likelihood of remaining married. Using a cross-sectional design, we tested whether gritty individuals were less likely to divorce.

Marital longevity is positively associated with emotional stability, agreeableness, and conscientiousness (Roberts et al., 2007). Associations between personality and marital status, however, are often moderated by gender. Big Five agreeableness, for example, is positively associated with relationship satisfaction among men, but not women (White et al., 2004). In light of these known gender-personality interactions, in Study 4 we examined the overall association between grit and marital status as well as whether this association was moderated by gender.

### Materials and methods

#### Participants

In total, 11,141 adults completed grit questionnaires online. Because we were interested in assessing the association between grit and the tendency, once married, to remain married, we excluded the 4648 (42%) participants who had never been married from further analyses. After also excluding the 2% of participants who did not complete requisite questionnaire items, our final sample consisted of *N* = 6362 (57%); *M* = 46.07 years, *SD* = 11.39. Eighty-seven percent of participants were White, 4% were Asian, 4% were Hispanic, 2% were Black, and 3% were of other ethnic backgrounds; 64% were female^2^. There were no significant differences between included vs. excluded participants on any study variables (*p* > 0.05).

#### Procedure and measures

Potential participants were directed to the online survey for this study via links on the last author's personal website and www.authentichappiness.org, a public psychology website.

***Retention: marital status***. Using a binary variable, participants were labeled as either 1 = *married* or 0 = *separated or divorced.*

***Grit***. The same grit measure described in Study 1 was used in the present study. The observed alpha was 0.79.

***Big Five personality traits***. The same personality scale described in Study 2 was used in the present study. Observed alphas ranged from 0.79 to 0.88.

### Results and discussion

Eighty percent of participants were married and 20% were separated or divorced. As shown in Table 4A, grit was strongly associated with Big Five conscientiousness (*r* = 0.71) and more modestly associated with other Big Five personality subscales (*r*s from 0.08 to 0.33) (see Table 4A). The grit scores of males (*M* = 3.47, *SD* = 0.69) were not significantly different than the grit scores of females (*M* = 3.47, *SD* = 0.70), *t*_(6, 360)_ = 0.14, *p* = 0.89.

**Table 4A:** **Summary Statistics and Intercorrelations for Married and Divorced participants (Study 4)**.

**Measures**	**Correlations^a^**					
	**1**	**2**	**3**	**4**	**5**	**6**
1. Grit	–					
2. Big Five extraversion	0.21^***^	–				
3. Big Five agreeableness	0.20^***^	0.21^***^	–			
4. Big Five conscientiousness	0.71^***^	0.18^***^	0.21^***^	–		
5. Big Five emotional stability	0.33^***^	0.30^***^	0.41^***^	0.32^***^	–	
6. Big Five openness to experience	0.08^***^	0.24^***^	0.14^***^	0.06^***^	0.11^***^	–
7. Female	−0.00	0.06^***^	0.11^***^	0.04^**^	−0.17^***^	0.04^**^
8. Age	0.12^***^	0.06^***^	0.14^***^	0.08^***^	0.10^***^	0.18^***^
9. White	−0.03^*^	0.01	0.04^**^	0.02	−0.02	0.04^***^
10. Asian	0.01	−0.02	−0.05^***^	−0.02	0.03^*^	−0.07^***^
11. Hispanic	0.02	0.00	−0.00	−0.02	−0.01	0.02
12. Black	0.02	0.02	0.02	0.01	0.03^*^	−0.00
13. Other	0.01	−0.01	−0.02	−0.01	−0.01	0.02
14. Some high school	−0.03^*^	0.01	0.01	−0.04^**^	0.02	−0.02
15. Finished high school	−0.02	−0.01	0.01	−0.02	−0.02	−0.04^***^
16. Some college	−0.06^***^	−0.00	0.02	−0.06^***^	−0.03^**^	−0.04^**^
17. Associate degree	−0.03^*^	−0.02	0.02	0.00	−0.03^*^	−0.01
18. Bachelor degree	−0.05^***^	−0.01	−0.02	−0.05^***^	−0.00	−0.03^*^
19. Post-college degree	0.11^***^	0.02	−0.01	0.09^***^	−0.04^**^	0.08^***^
Observed range	1.00–5.00	1.00–5.00	1.11–5.00	1.00–5.00	1.00–5.00	1.30–5.00
*M*	3.47	3.41	3.85	3.81	3.26	4.03
*SD*	0.70	0.85	0.62	0.68	0.84	0.61

N = 6362.

* p < 0.05;

** p < 0.01;

*** p < 0.001.

a Full correlations among demographics are truncated to conserve space.

As shown in Table 4B, in separate binomial logistic regression models, Big Five conscientiousness (*OR* = 1.08) was associated with an increase in the likelihood of remaining married, whereas agreeableness (*OR* = 0.87) and openness to experience (*OR* = 0.76) were associated with decreases in the likelihood of remaining married. Grit, extraversion and emotional stability were not significantly associated with marital status. Women were less likely to remain married (*OR* = 0.33) as were older participants (*OR* = 0.55), participants who attended some college but did not earn a degree (*OR* = 0.61), and participants with an associate's degree (*OR* = 0.57). By contrast, Asians were more likely to remain married (*OR* = 2.26), as were participants with a Bachelor's degree (*OR* = 1.19).

**Table 4B:** **Bivariate and full logistic regressions predicting tendency to remain married (Study 4)**.

**Measures**	**Bivariate model**			**Full model**		
	***OR***	**95% CI**	***% R*^2^^a^**	***OR***	**95% CI**	***% R*^2^^a^**
Grit	1.04	[0.98, 1.11]	0.00	–	–	–
Grit (for females)	–	–	–	0.95	[0.86, 1.04]	–
Grit (for males)	–	–	–	1.17^*^	[1.00, 1.36]	–
Big Five extraversion	0.96	[0.90, 1.02]	0.04	–	–	–
Big Five agreeableness	0.87^***^	[0.82, 0.92]	0.25	0.99	[0.93, 1.06]	0.00
Big Five conscientiousness	1.08^*^	[1.02, 1.15]	0.29	1.18^**^	[1.07, 1.29]	0.27
Big Five emotional stability	1.05	[0.98, 1.11]	0.00	–	–	–
Big Five openness to experience	0.76^***^	[0.71, 0.81]	1.78	0.81^***^	[0.76, 0.87]	0.81
Female	0.33	[0.28, 0.38]	5.80	0.34^***^	[0. 29, 0.40]	–
Age	0.55^***^	[0.51, 0.60]	6.22	0.55^***^	[0.51, 0.60]	5.00
Asian	2.26	[1.54, 3.32]	0.51	1.19	[0.80, 1.77]	0.02
Hispanic	0.85	[0.62, 1.15]	0.00	0.73	[0.53, 1.01]	0.08
Black	0.83	[0.52, 1.31]	0.02	0.64	[0.39, 1.04]	0.06
Other	0.86	[0.62, 1.18]	0.02	0.73	[0.52, 1.03]	0.07
Some high school	0.64	[0.34, 1.23]	0.04	0.45^*^	[0.23, 0.91]	0.10
Finished high school	0.96	[0.62, 1.48]	0.00	0.81	[0.51, 1.29]	0.02
Some college	0.61^***^	[0.51, 0.73]	0.69	0.58^***^	[0.48, 0.71]	0.00
Associate degree	0.57^***^	[0.44, 0.74]	0.42	0.56^***^	[0.43, 0.74]	0.34
Bachelor degree	1.19^*^	[1.04, 1.36]	0.16	0.92	[0.79, 1.07]	0.03

N = 6362.

* p < 0.05;

** p < 0.01;

*** p < 0.001.

a The Nagelkerke index was used to compute Pseudo R^2^.

**Figure 1:**
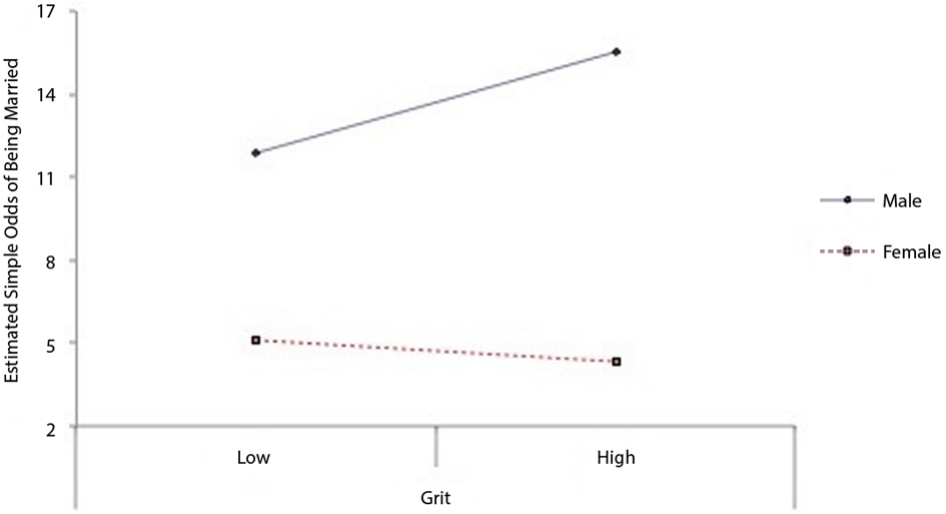
**Estimated simple odds of being married vs. divorced as a function gender and grit.** Low (−1 *SD*) and high (+1 *SD*) grit are displayed on the x-axis. Control variables include gender, age, race, level of education, Big Five agreeableness, Big Five conscientiousness, and Big Five openness to experience.

Given that grit was not a significant predictor of retention in the bivariate model, it is unsurprising that in the full model, grit was not associated with marital status. In order to test whether the relation between grit and remaining married varied by gender, we tested another model. In this model we included a term representing the interaction between grit and gender. This model revealed a significant interaction between grit and gender such that grit was associated with 17% increased odds of remaining married among men, but was not associated with greater odds of remaining married among women (see Table 4B)^3^. Figure 1 shows the estimated simple odds of being married at high (+1 *SD*) and low (−1 *SD*) levels of grit for men and women respectively. In this final model, participants higher in conscientiousness were also more likely to be married (*OR* = 1.18), whereas participants higher in openness to experience (*OR* = 0.81) were less likely to have remained married. Examining the unique variance explained by each predictor in the final model, the only other non-demographic variables to explain unique variance in marital longevity were conscientiousness (Δ*R*^2^ = 0.27%) and openness to experience (Δ*R*^2^ = 0.81%): participants who self-rated higher in conscientiousness were more likely to remain married whereas participants who self-rated higher in openness to experience were less likely to remain married.

Next we ran a hierarchical logistic regression to confirm the unique predictive validity of the Grit × Gender interaction over and beyond all other predictors. All predictor variables except the Grit × Gender interaction were entered in Step 1 of this model. The Grit × Gender interaction was entered in Step 2, as shown in Table 4B. Results of the hierarchical logistic regression revealed that Step 2 contributed significantly to the model, χ^2^_(1)_ = 7.25, *p* < 0.007.

We then ran an additional series of models compare the effect of a Grit × Gender interaction to a Conscientiousness × Gender interaction. First, we tested a model identical to the full model in Table 4B but for the substitution of a Conscientiousness × Gender interaction for the Grit × Gender interaction. This model revealed a significant interaction of nearly identical magnitude. In a subsequent model that included both the Grit × Gender interaction as well as the Conscientiousness × Gender interaction, neither interaction reached significance. These findings suggest that the Conscientiousness × Gender and Grit × Gender interactions explain overlapping variance in the retention outcome. As a result, when these two interactions are entered in the model together, neither explains unique variance in retention.

The most intriguing finding of Study 4 was that grit—and conscientiousness—correlated with marital status among men but not women. Due to the cross-sectional nature of this investigation, as well as the fact that only one member of each couple was surveyed, we can only speculate as to why the association between grit and marital status was gender-specific. One possibility is that men find it harder to remain married to women than the obverse, and therefore grit predicts marital status among men but not women. Additional research is needed to illuminate the differential mechanisms underlying the association between grit and marital status for men vs. women. It is also important to qualify this finding in light of past research on conscientiousness, gender, and marital longevity. Although the association between grit and marital longevity has not been studied to date, in past research, conscientiousness demonstrates small negative associations with divorce among both men and women (*r*s ranging from −0.07 to −0.12; Kurdek, 1993; Tucker et al., 1998; for review see Roberts et al., 2007).

Overall, the results indicated that grittier men were more likely to be married than separated or divorced, but there was no association between grit and marital status among women. Notably, the effect of grit on marital status among men held when controlling for Big Five personality traits and demographic covariates.

### General discussion

Across four studies, grittier individuals were less likely to drop out of their respective life commitments: Gritty soldiers were more likely to complete 3 weeks of a grueling ARSOF selection course (Study 1), gritty sales representatives were more likely to remain at their jobs three months later (Study 2), gritty high school juniors were more likely to graduate from high school 1 year later (Study 3), and gritty men (but not women) were more likely to remain married (Study 4). Taken together, these findings take the first step toward establishing the association between grit and persistence across a range of life contexts. Overall, grit and other measured individual differences each explained small, approximately comparable amounts of variance in the retention outcome. These findings are consistent with past research showing that personality traits have small or small-to-medium sized predictive validity over and above individual difference variables and demographic predictors of life outcomes (Roberts et al., 2007).

The results of the current studies enrich what is known about the association between grit and retention by showing that grit is associated with retention not only in high-achieving populations (e.g., cadets at West Point; Duckworth et al., 2007) but also among sales representatives (Study 2), and juniors in the Chicago Public Schools (Study 3). The association between grit and marital longevity (Study 4) shows that grit is predictive of retention even outside the traditional “achievement” context, a context in which grit had not previously been examined.

### Limitations

The four studies in this paper have a number of limitations. First, due to the correlational nature of the present studies, it cannot be inferred that grit was causally related to retention.

Second, the Grit Scale, like all self-report scales, is vulnerable to social desirability bias (e.g., Lucas and Baird, 2006). Mitigating this concern is the fact that grit was associated with retention after controlling for other self-reported measures (e.g., Big Five personality traits), which would have been also been affected by social desirability bias. Also mitigating this concern is the fact that mean grit scores across samples conformed with what might be expected in reality: ARSOF candidates (Study 1) and sales representatives (Study 2) evinced higher average grit scores than students in the Chicago Public schools (Study 3) and adults recruited to participate in a marriage study on the internet (Study 4), two less selective samples.

A third limitation is that conscientiousness was only included as a covariate in Study 2 and Study 4. Future studies measuring the association between grit and retention should systematically measure conscientiousness. Moreover, due to time considerations, a full battery of conscientiousness-level subscales could not be included in the present studies. Future work should include facets of conscientiousness, including orderliness, self-control, and industriousness. Such comprehensive multi-trait studies would more stringently test the incremental predictive validity of grit.

Another major limitation is that retention, as measured in the present research, was not necessarily in line with the individual's subjective goals. Commitment to retention goals was assumed but not measured directly. It therefore remains possible that individuals who dropped out held other goals (e.g., to make money now) that conflicted with assumed retention goals (e.g., to graduate from this high school). Indeed, it is possible that in such cases grit would be manifest by abandoning one's current training program, job, school program, or marriage for a better one. Additional research is needed in which grit is studied as a predictor of short-term retention goals as well as long-term success and satisfaction.

Finally, in order to establish grit as a domain-general trait, future research ought to examine the association between grit and participants' actions across a host of domains. The present investigation, which assessed grit among different participants in different settings, does not fully illuminate whether individuals who renege on their commitments in one life domain (e.g., school) are likely to also do so in other areas (e.g., marriage).

## Conclusion

Consistent with the theoretical model proposed by Glick and Carter (1958) over five decades ago, the findings of the present investigation suggest that a personality trait contributes to dropping out of commitments. Whereas subsequent research largely abandoned Glick's hypothesis in search of situational determinants of school and marital dropout (Bauman, 1967; Glenn and Supancic, 1984), our research redirects efforts to understand sustained commitments back to the psychological domain. Future investigations should explore related topics: Are there conditions under which grit is less predictive of retention and accomplishment? What can be done to intentionally cultivate grit? How do domain-general variables like grit interact with domain-specific factors to determine how long individuals remain committed to their pursuits? To the extent that we can enhance people's abilities to pursue their interests with vigor and persistence, we may improve their life prospects and, ultimately, their well-being.

## Author note

We thank Sigal Barsade, Nancy Rothbard, and Ari Lustig for their help in conducting this investigation. This research was supported by funding from the National Institutes of Health (5-K01-AG033182-02) and the Character Lab.

### Conflict of interest statement

The authors declare that the research was conducted in the absence of any commercial or financial relationships that could be construed as a potential conflict of interest.

## Footnotes

^1^As a conservative test of our findings, we re-ran all analyses in Study 1 including these excluded participants. The inclusion of these participants had no noticeable effect on the reported results.

^2^Past research suggests that Whites (Voigt et al., 2003), females (Moore and Tarnai, 2002) and individuals with higher education (Curtin et al., 2000) are more likely to participate in surveys, which may explain the slightly skewed demographic distribution in the present study.

^3^The grit by gender interaction was examined retrospectively in each of the other studies in this investigation. It was non-significant in Studies 1, 2, and 3 (*p* > 0.05).
