# Supplementary material for: The grit effect: predicting retention in the military, the workplace, school and marriage
Source: Front Psychol. 2014 Feb 3;5:36. doi: 10.3389/fpsyg.2014.00036 (PMC3910317; doi:10.3389/fpsyg.2014.00036)
Supplement: Supplementary file 1 [file DataSheet1.PDF]

SUPPLEMENTARY MATERIAL

The grit effect: predicting retention in the military, the workplace, school and marriage

Lauren Eskreis-Winkler1*, Elizabeth P. Shulman1, Scott A. Beal2, Angela Lee Duckworth11Psychology, University of Pennsylvania, Philadelphia, PA, USA2Fort Bragg Research Element, U.S. Army Research Institute, VA, USA

Correspondence concerning this article can be addressed to Lauren Eskreis-Winkler, Department of Psychology, University of Pennsylvania, 3701 Market Street, Suite 207, Philadelphia, PA 19104. E-mail: eskreisl@sas.upenn.edu.

Table 1a. Summary Statistics and Intercorrelations among ARSOF Candidates (Study 1).

N = 677.

s	Correlations[a]			
Variables	1	2	3	4
1. Grit				
2. General intelligence	-.07			
3. Physical fitness	.06	.09*		
4. Age	.12**	-.05	-.13**	
5. Years of schooling	-.00	.42***	.11**	.24***

Note. *$p < .05$. **$p < .01$. ***$p < .001$.

[a] Full correlations among demographics are truncated to conserve space. See Appendix A for full details.

Table 2a. Summary Statistics and Intercorrelations for Sales Employees (Study 2). *N* = 442.

	Correlations[a]					
Variables	1	2	3	4	5	6
1. Grit						
2. Big Five extraversion	.25***					
3. Big Five agreeableness	.39***	.26***				
4. Big Five conscientiousness	.64***	.37***	.53***			
5. Big Five emotional stability	.48***	.30***	.44***	.53***		
6. Big Five openness to experience	.19***	.34***	.30***	.33***	.31***	
7. Female	-.02	.09	.11*	.05	-.08	.05
8. Age	.20***	.00	.15**	.17***	.21***	.02
9. White	-.10*	.07	-.01	-.02	-.15**	-.09
10. Black	.10*	-.06	.01	.01	.12*	.04
11. Hispanic	-.01	-.03	-.01	-.02	.08	.07
12. Asian	.04	-.03	.02	.04	.02	.04
13. Other	.03	-.01	.02	.03	-.01	.04
14. Weeks employed	.08	.03	-.01	.06	-.05	-.02
15. Years in sales	.15**	.08	.08	.12*	.09	.06

Note. *$p < .05$. **$p < .01$. *** $p < .001$

Table 2a, continued. Summary Statistics and Intercorrelations for Sales Employees (Study 2). $N = 442$.

	Correlations[a]							
Variables	7	8	9	10	11	12	13	14
1. Grit								
2. Big Five extraversion								
3. Big Five agreeableness								
4. Big Five conscientiousness								
5. Big Five emotional stability								
6. Big Five openness to experience								
7. Female								
8. Age	-.08							
9. White	.13**	.12*						
10. Black	-.14**	-.05	-.56***					
11. Hispanic	-.03	-.06	-.49***	-.08				
12. Asian	-.07	-.04	-.23***	-.04	-.03			
13. Other	.03	-.07	-.43***	-.08	-.07	-.02		
14. Weeks employed	-.05	.10*	-.08	-.04	.04	-.09	.18***	
15. Years in sales	-.04	.51***	.06	-.02	-.00	.03	-.05	-.04

Note. *$p < .05$. **$p < .01$. *** $p < .001$.

Table 3a. Summary Statistics and Intercorrelations for Chicago Public School students (Study 3). $N = 4{,}813$

	Correlations[a]							
Measures	1	2	3	4	5	6	7	8
1. Grit								
2. Standardized achievement tests	.15***							
3. Academic conscientiousness	.49***	.06***						
4. School motivation	.49***	.12***	.50***					
5. Perceived school safety	.15***	.28***	.13***	.16***				
6. Perceived teacher support	.38***	.12***	.51***	.55***	.26***			
7. Perceived parental support	.34***	.07***	.29***	.30***	.10***	.27***		
8. Perceived peer support	.42***	.13***	.48***	.53***	.19***	.53***	.27***	
9. Female	.14***	-.01	.13***	.14***	-.08***	.09***	.06***	.15***
10. Hispanic	-.08***	.04*	-.09***	-.03*	.02	.00	-.05**	-.07***
11. Black	.07***	-.20***	.06***	.03	-.11***	-.03	.06***	.06***
12. White	.00	.15***	.02	-.01	.10***	.00	.02	-.03
13. Asian	.02	.20***	.05**	.03*	.09***	.06***	-.05**	.04**
14. Other	.03*	.01	.01	.01	.02	.00	-.00	.01
15. Free lunch	-.01	-.18***	-.02	-.01	-.07***	-.03*	-.03	-.03
16. Reduced price lunch	.00	.09***	.02	.01	.03*	.01	.01	.01
17. Full price lunch	-.00	.15***	.01	-.01	.07***	.03*	.02	.02

Note. $^{*}p < .05$. $^{**}p < .01$. $^{***}p < .001$.

Table 3a, continued. Summary Statistics and Intercorrelations for Chicago Public School students (Study 3). $N = 4,813$.

	Correlations[a]							
Measures	9	10	11	12	13	14	15	16
1. Grit								
2. Standardized achievement tests								
3. Academic conscientiousness								
4. School motivation								
5. Perceived school safety								
6. Perceived teacher support								
7. Perceived parental support								
8. Perceived peer support								
9. Female								
10. Hispanic	-.03							
11. Black	.05**	-.80***						
12. White	-.02	-.24***	-.23***					
13. Asian	-.01	-.20***	-.19***	-.06***				
14. Other	.01	-.03*	-.03	-.01	-.01			
15. Free lunch	.03*	.03*	.09***	-.18***	-.06***	.00		
16. Reduced price lunch	-.01	.03	-.07***	.08***	.02	.01	-.76***	
17. Full price lunch	-.03*	-.07***	-.04**	.17***	.07***	-.01	-.57***	-.12***

Note. *$p < .05$. **$p < .01$. *** $p < .001$.

Table 4a. Summary Statistics and Intercorrelations for Married and Divorced participants (Study 4). $N = 6,362$.

	Correlations[a]						
Measures	1	2	3	4	5	6	7
1. Grit							
2. Big Five extraversion	.21***						
3. Big Five agreeableness	.20***	.21***					
4. Big Five conscientiousness	.71***	.18***	.21***				
5. Big Five emotional stability	.33***	.30***	.41***	.32***			
6. Big Five openness to experience	.08***	.24***	.14***	.06***	.11***		
7. Female	-.00	.06***	.11***	.04**	-.17***	.04**	
8. Age	.12***	.06***	.14***	.08***	.10***	.18***	.07***
9. White	-.03*	.01	.04**	.02	-.02	.04***	.05***
10. Asian	.01	-.02	-.05***	-.02	.03*	-.07***	-.07***
11. Hispanic	.02	.00	-.00	-.02	-.01	.02	.01
12. Black	.02	.02	.02	.01	.03*	-.00	-.01
13. Other	.01	-.01	-.02	-.01	-.01	.02	-.01
14. Some high school	-.03*	.01	.01	-.04**	.02	-.02	-.02
15. Finished high school	-.02	-.01	.01	-.02	-.02	-.04***	.01
16. Some college	-.06***	-.00	.02	-.06***	-.03**	-.04**	.07***
17. Associate degree	-.03*	-.02	.02	.00	-.03*	-.01	.07***
18. Bachelor degree	-.05***	-.01	-.02	-.05***	-.00	-.03**	-.04**
19. Post-college degree	.11***	.02	-.01	.09***	-.04**	.08***	-.04**

Note. *$p < .05$. **$p < .01$. *** $p < .001$

Table 4a, continued. Summary Statistics and Intercorrelations for Married and Divorced participants (Study 4). $N = 6,362$.

Note. *$p < .05$. **$p < .01$. *** $p < .001$

Measures	Correlations						
	8	9	10	11	12	13	14
1. Grit							
2. Big Five extraversion							
3. Big Five agreeableness							
4. Big Five conscientiousness							
5. Big Five emotional stability							
6. Big Five openness to experience							
7. Female							
8. Age							
9. White	.15***						
10. Asian	-.13***	-.55***					
11. Hispanic	-.05***	-.50***	-.05**				
12. Black	-.04**	-.33***	-.03*	-.03*			
13. Other	-.05***	-.49***	-.04**	-.04**	-.02		
14. Some high school	-.02	-.02	.00	.01	.02	.02	
15. Finished high school	-.01	.01	-.02	.00	-.02	.02	-.01
16. Some college	-.02	.01	-.05***	.02	.01	.00	-.03*
17. Associate degree	.01	.03**	-.04**	.01	-.01	-.02	-.02
18. Bachelor degree	-.09***	.01	-.02	.00	.01	.00	-.06***
19. Post-college degree	.09***	-.03*	.07***	-.02	-.01	-.01	-.09***

Table 4a, continued. Summary Statistics and Intercorrelations for Married and Divorced participants (Study 4). $N = 6{,}362$.

	Correlations			
Measures	15	16	17	18
1. Grit				
2. Big Five extraversion				
3. Big Five agreeableness				
4. Big Five conscientiousness				
5. Big Five emotional stability				
6. Big Five openness to experience				
7. Female				
8. Age				
9. White				
10. Asian				
11. Hispanic				
12. Black				
13. Other				
14. Some high school				
15. Finished high school				
16. Some college	-.05***			
17. Associate degree	-.03*	-.08***		
18. Bachelor degree	-.09***	-.23***	-.15***	
19. Post-college degree	-.14***	-.36***	-.23***	-.68***

Note. *$p < .05$. **$p < .01$. *** $p < .001$
